# High survivorship after catch-and-release fishing suggests physiological resilience in the endothermic shortfin mako shark (*Isurus oxyrinchus*)

**DOI:** 10.1093/conphys/cov044

**Published:** 2015-09-30

**Authors:** Robert P French, Jeremy Lyle, Sean Tracey, Suzanne Currie, Jayson M Semmens

**Affiliations:** af1 Fisheries and Aquaculture Centre, Institute for Marine and Antarctic Studies, University of Tasmania, Hobart, Tasmania 7001, Australia; af2 Department of Biology, Mount Allison University, Sackville, New Brunswick, Canada E4L 1E4

**Keywords:** Catch-and-release fishing, endothermy, mako shark, post-release survival, stress physiology

## Abstract

The shortfin mako shark (*Isurus oxyrinchus*) is a species commonly targeted by commercial and recreational anglers in many parts of the developed world. In Australia, the species is targeted by recreational anglers only, under the assumption that most of the sharks are released and populations remain minimally impacted. If released sharks do not survive, the current management strategy will need to be revised. Shortfin mako sharks are commonly subjected to lengthy angling events; however, their endothermic physiology may provide an advantage over ectothermic fishes when recovering from exercise. This study assessed the post-release survival of recreationally caught shortfin mako sharks using Survivorship Pop-up Archival Transmitting (sPAT) tags and examined physiological indicators of capture stress from blood samples as well as any injuries that may be caused by hook selection. Survival estimates were based on 30 shortfin mako sharks captured off the south-eastern coast of Australia. Three mortalities were observed over the duration of the study, yielding an overall survival rate of 90%. All mortalities occurred in sharks angled for <30 min. Sharks experienced increasing plasma lactate with longer fight times and higher sea surface temperatures (SSTs), increased plasma glucose at higher SSTs and depressed expression of heat shock protein 70 and β-hydroxybutyrate at higher SSTs. Long fight times did not impact survival. Circle hooks significantly reduced foul hooking when compared with J hooks. Under the conditions of this study, we found that physical injury associated with hook choice is likely to have contributed to an increased likelihood of mortality, whereas the high aerobic scope associated with the species' endothermy probably enabled it to cope with long fight times and the associated physiological responses to capture.

## Teaser

We used satellite tags and blood based analyses to examine the impacts of catch and release angling on the shortfin mako shark. We report high survivorship seemingly unaffected by fight time or physiological stress and recommend that circle hooks should be used to reduce physical damage to these sharks.

## Introduction

Recreational fishing is a popular pastime in many parts of the developed world (Post *et al.*, 2002) and, while the negative impacts of fishing on global populations have typically been attributed to commercial fisheries, it is becoming more commonly accepted that the recreational sector also contributes to many of these impacts (McPhee *et al.*, 2002; Post *et al.*, 2002; Coleman *et al.*, 2004; Cooke and Cowx, 2004; Arlinghaus *et al.*, 2005; Lewin *et al.*, 2006). For decades, catch-and-release fishing methods have been advocated by fisheries managers and recreational fishing organizations in an attempt to promote the sustainable use of fisheries resources (Policansky, 2002; Arlinghaus *et al.*, 2007). However, it is recognized that not all individuals are likely to survive once released, with post-release survival rates being highly variable among species (Muoneke and Childress, 1994; Bartholomew and Bohnsack, 2005; Skomal and Mandelman, 2012; Gallagher *et al.*, 2014). This highlights the need to assess post-release survival on a species-by-species basis.

Like many elasmobranchs, the shortfin mako shark (*Isurus oxyrinchus*) is vulnerable to fishing pressure because of its life-history characteristics (Hoenig and Gruber, 1990; Stevens, 2008; Semba *et al.*, 2011). It is an endothermic species, with one of the highest metabolic rates recorded for any pelagic shark; this implies a high aerobic scope that could be an advantage when dealing with physiological disturbances (Sepulveda *et al.*, 2007). The shortfin mako shark is a popular target species for game fishers and a substantial portion of the bycatch in commercial longline fisheries targeting tuna and billfish (Stevens, 2008). In Australia, the shortfin mako shark was listed as a protected species under the Environment Protection and Biodiversity Conservation (EPBC) Act in 2010, following listings by the International Union for Conservation of Nature (IUCN) and Convention on the Conservation of Migratory Species (CMS), as ‘vulnerable’ and ‘migratory’, respectively. A controversial political debate around the protection of the species in Australia resulted in the shortfin mako shark remaining available to be targeted by recreational anglers only, under the assumption that most of the sharks are released and that populations remain minimally impacted by the fishery. There is, however, little information on post-release survival rates for recreationally caught shortfin mako sharks, and thus uncertainty as to the efficacy of the current management strategy.

In many instances, angling mortality can be linked to physical injuries associated with the gear used and the handling of the animal (Muoneke and Childress, 1994; Cooke and Hogle, 2000; Cooke and Suski, 2004; Bartholomew and Bohnsack, 2005; Campana *et al.*, 2009; Carruthers *et al.*, 2009; Burns and Froeschke, 2012). Capture-related physiological disruptions exceeding a fish's ability to return to homeostasis can also result in mortality of released individuals (Kieffer, 2000; Moyes *et al.*, 2006; Hight *et al.*, 2007; Frick *et al.*, 2010, 2012). Additionally, physiological disruptions can influence the behaviour of released fish, resulting in increased vulnerability to predation during the recovery period (Brownscombe *et al.*, 2014; Raby *et al.*, 2014).

Physiological responses to stressors can be observed through changes in blood chemistry, and it has been observed that the magnitude of the stress response in sharks and other fishes can be linked to environmental factors, such as water temperature (Kieffer *et al.*, 1994; Manire *et al.*, 2001). These responses include the anaerobic breakdown and mobilization of energy reserves, such as glucose and glycogen, to meet energetic demands and the associated accumulation of lactate (La^−^) and metabolic protons (H^+^), leading to lacticacidosis (Skomal and Mandelman, 2012). Changes in plasma ion concentrations can also result from lacticacidosis and drive cellular fluid shifts that result in haemoconcentration and disruptions to osmotic homeostasis (Skomal and Mandelman, 2012). A cellular stress response involving heat shock proteins (HSPs) may also be present if cellular proteins are negatively impacted by the stress (Roberts *et al.*, 2010; Currie, 2011). Recovery from these impacts is an aerobic process fuelled partly by the oxidation of ketones (Richards *et al.*, 2003), and it has been suggested that interspecific differences in dealing with capture stress may be linked, in part, to the metabolic scope and thermal physiology of the target species (Skomal and Bernal, 2010; Skomal and Mandelman, 2012). Therefore, in understanding the implications of capture on subsequent survival it is necessary to screen for a suite of physiological and cellular markers (Skomal, 2007).

Post-release survival itself can be problematic to assess, particularly in large migratory species, where controlled experiments are not possible and conventional tag recapture studies may be limited by dispersal (Moyes *et al.*, 2006; Skomal, 2007). Satellite tags are one way of addressing survivorship in large migratory animals (Graves *et al.*, 2002; Stokesbury *et al.*, 2011); however, the cost of tags often precludes large sample sizes (Donaldson *et al.*, 2008). The recent development of specialized survivorship tags provides researchers with a more cost-effective solution to this problem (Hutchinson *et al.*, 2015).

This study aimed to quantify the post-release survival rate for recreationally caught shortfin mako sharks, with consideration of the nature and magnitude of the physiological response to capture. Given the high metabolic rate and aerobic scope associated with this species' thermal strategy, we hypothesized a high post-release survival rate that is independent of the level of physiological stress experienced during recreational capture.

## Materials and methods

### Capture and sampling

Shortfin mako sharks were caught in south-eastern Australian waters off the coast of Tasmania (Tas), South Australia (SA) and New South Wales (NSW) using gears and methods commonly used by Australian game fishers when targeting this species. Sharks were attracted to the boat using chum and offered a baited hook once sighted. Each shark was allowed to take the bait and swim away from the boat before the hook was set. Gear used included 15, 24 and 37 kg rated monofilament line, joined to a ∼130 kg monofilament wind-on leader and 1.6 mm stainless-steel wire trace. Terminal tackle alternated between non-offset Shogun 9/0 stainless-steel J (straight shank) hooks and non-offset Eagle Claw 13/0 circle hooks. Once boat-side, 26 sharks were left in the water and restrained by looping a thick, soft rope around the body posterior to the pectoral fins. Fight time (time from hook-up to restraint) was recorded to the nearest minute. The boat was kept in gear and the shark moved slowly forward, facilitating ventilation of the gills. Seven sharks were manually lifted through a dive door for handling on deck; once where the tag applicator would not penetrate the skin, and six times when small (<∼50 kg) sharks either became tangled in gear or when it was deemed more efficient to handle them without the use of rope. In such instances, animals were not ventilated in order to replicate game fishing conditions as closely as possible; no restraint on deck was necessary.

Once restrained or on deck, a pre-heparinized 5 ml syringe fitted with a 16 gauge needle was used to take a ∼4 ml blood sample via caudal puncture. The sharks were measured to the nearest centimetre (fork length) and sex and hooking location (Table 1) noted. Sharks were then tagged adjacent to the dorsal fin with a Survivorship Pop-up Archival Transmitting (sPAT) tag (Wildlife Computers) fitted with a Domeier umbrella anchor. Where possible, the hook was removed before release; if this was not possible, the trace was cut as close to the hook as possible. Each shark was examined for physical damage associated with hooking and substantial bleeding (free-flowing blood that was not obviously slowed by natural haemostasis) noted. Handling time (time from restraint to release) was recorded to the nearest minute, and the general condition upon release and the vigour of the shark as it swam off were also categorized (Table 1).

**Table 1:** Detailed definitions of variables recorded from each shark at capture

Hooking locations	Jaw	Hooked around the jaw directly, including gums
	Throat	Hook set behind teeth to oesophagus, excluding gill arches or filaments. Hook still visible
	Gills	Hook set internally in gill arches or gill filaments
	Gut	Hook set in deep oesophagus (beyond vision) and further down alimentary canal
	Body	Hook set in any external surface of the shark, excluding jaw
Condition at capture	Good	Active and responsive shark with no damage beyond the hook puncture
	Average	Shark appears exhausted, is not very responsive or has sustained superficial injuries
	Poor	Shark appears dead or dying (moribund) or has sustained heavy injuries/heavy bleeding
Swimming vigour at release	Strong	Vigorous or high-energy swimming
	Well	Regular pre-capture-like swimming
	Slow	Exhausted, sluggish or buoyancy troubled
	Lifeless	No active swimming at all; drifted away

### Post-release survival

Post-release survival was determined using data from the sPAT tags as per Hutchinson *et al.* (2015). These tags are pre-programmed to release and report survival after 30 days at liberty or to report prematurely if mortality occurs. Each tag summarizes data *in situ* and transmits daily minimal and maximal temperature and depth, light change (day–night transitions) and attachment pin status (whether or not the tag has separated from the anchor). These data, along with the final pop-up location, are transmitted via Argos satellites once the tag reaches the surface and are used to determine whether the shark was actively swimming and alive at the time of release. If no movement (no depth change) is detected over 24 h or the tag exceeds 1700 m in depth, the tag will release prematurely. The fate of each tag, and therefore each shark, will fall into one of the following four categories: ‘completed deployment’, ‘sinker’, ‘sitter’ or ‘floater’. A ‘completed deployment’ refers to a tag still attached to a swimming animal 30 days after deployment; from this, survival is inferred as recovery from any physiological disturbances associated with the capture experience is expected to have occurred well within this deployment period (Frick *et al.*, 2010). ‘Sinker’ is assigned to tags that surpass 1700 m in depth, it can be assumed that the tag is attached to a shark that has died and is sinking; this depth is well beyond the maximum reported for shortfin mako sharks (Abascal *et al.*, 2011). ‘Floater’ refers to a tag that remains floating on the surface for 24 h; this may indicate attachment failure or possible fishing mortality. ‘Sitter’ refers to a tag remaining at a constant depth that is shallower than 1700 m for 24 h, implying that the shark died, sank and is resting on the ocean floor.

### Biochemical analyses

Whole-blood glucose and lactate were quantified immediately with the use of hand-held meters [Accu-Chek Active blood glucose meter (Roche); Lactate Pro (Arkray)]. Spun haematocrit was determined by centrifuging blood for 5 min at a relative centrifugal force of 4400***g*** in duplicate 75 mm mylar-coated capillary tubes plugged with Critoseal clay (ZIPocrit portable haematocrit centrifuge; LW Scientific). The remaining blood was then centrifuged at a relative centrifugal force of 2800***g*** for 5 min (ZIPspin microcentrifuge; LW Scientific) to separate plasma and red blood cells (RBCs), and immediately placed in liquid nitrogen for later analysis using the procedures detailed below. Long-term storage was at −80°C.

#### Protein levels of heat shock protein 70

Soluble protein was extracted from RBCs as per LeBlanc *et al.* (2012). Each sample was diluted in 200 µl of shark saline (mM: 280 NaCl; 7 KCl; 10 CaCl_2_; 4.9 MgCl_2_; 8 NaHCO_3_; 1 NaH_2_PO_4_; 0.5 Na_2_SO_4_; pH 7.8; modified from Villalobos and Renfro, 2007) before DNA was sheared. This saline was free of urea, trimethylamine oxide (TMAO) and glucose for analysis purposes, because these compounds were measured as part of the experimental protocol. The resulting supernatant was diluted 1:200 in shark saline and assayed (BioRad) at 750 nm using a VERSA_MAX_ microplate reader.

Heat shock protein 70 was analysed based on methods developed for spiny dogfish (*Squalus acanthias*; Kolhatkar *et al.*, 2014). Thirty micrograms of soluble protein was run alongside a four-point standard curve (5, 15, 45 and 135 ng) of HSP70/72 standard (SPP-758; Enzo Life Sciences). The primary antibody AS05-083A diluted 1:4000 in ECL (Global anti-HSP70; Agrisera; recognizing both constitutive and inducible isoforms of HSP70). Imaging was captured in a VERSA_DOC_™ imaging system (MP 4000; BioRad) with Quantity One software. Image Lab^®^ software (BioRad) was used to quantify the band signal against the standard curve.

#### Plasma lactate

Given that the Lactate Pro is designed for use on human samples, we used a plasma lactate assay to validate the results obtained from the hand meter. Plasma lactate was quantified using a NADH-linked spectrophotometric assay as described by Currie *et al.* (1999). Samples were incubated for 30 min after the addition of the glycine buffer before concentrations were read at 340 nm using a VERSA_MAX_ microplate reader.

The values obtained from the Lactate Pro meter were tested for agreement against lactate assay values using Bland–Altman analysis (Bland and Altman, 1995, 1999; Krouwer, 2008). Given that a number of assay samples were compromised by equipment malfunction, this procedure allowed the use of a larger, more accurate data set with respect to lactate concentrations. Data were log transformed to account for normality and proportional error of differences during the analysis. Transformed Lactate Pro values under-represented the assay values by a mean difference of −0.4119 (*P* = 0.007; *n* = 30). These values were adjusted accordingly by adding 0.4119 and converted back to reflect untransformed values. The adjusted lactate values were used in all further statistical analyses.

#### Plasma ions

Na^+^, Cl^−^ and K^+^ were quantified by diluting plasma samples 1:2 with double-distilled H_2_O and analysing with a Diamond Diagnostics SmartLyte electrolyte analyser.

#### Osmolytes

Red blood cell and plasma urea were measured in accordance with Kolhatkar *et al.* (2014). The saline described above (see ‘*Protein levels of heat shock protein 70*’) was used for dilutions. Plasma TMAO was analysed by diluting samples 1:5 in cold acetone before analysis using a quadrupole linear ion trap (LTQ) mass spectrometer as described by MacLellan *et al.* (2015). Five microlitres of sample was diluted in 495 µl of 50/49.9/0.1 (v/v/v) methanol/water/formic acid; this solution was then laced with 5 µl of d9-TMAO (0.01 M) to give a final concentration of 0.1 mM. Ten microlitres of sample was injected into the mass spectrometer in triplicate. Plasma TMAO concentrations were determined by comparing the signal strengths of both endogenous and labelled TMAO and applying the appropriate dilution factor.

#### β-Hydroxybutyrate

Plasma β-hydroxybutyrate (β-OHB) was quantified using a colorimetric assay kit (Cayman Chemical Company IN: 700190) with a VERSA_MAX_ microplate reader according to the manufacturer's instructions.

### Statistical analyses

The 95% confidence interval associated with the survival estimate was calculated using the Release Mortality version 1.1.0 software developed by Goodyear (2002) and based on 10,000 simulations with no error sources or natural mortality incorporated, as described by Kerstetter and Graves (2006).

All other statistical analyses were carried out using SPSS (IBM) and R (R Core Team, 2014). A Kruskal–Wallis H test was used to determine whether the size distributions of sharks differed between sampling regions (NSW, Tas and SA) and whether concentrations of blood parameters differed between sharks with fight times <70 min (the bulk of the data) and four sharks with fight times that exceeded this limit (122–513 min). The non-parametric tests were chosen because a Sharpio–Wilk test of normality indicated that non-normal distributions were present in these data. Adjusted *P*-values are presented. The association between hook type and the occurrence of foul hooking (throat, gut, body and gill locations combined) vs. jaw hooking was investigated using a χ^2^ test. Fisher's exact test was used to determine whether two uncontrolled components of our handling procedure contributed to mortality, i.e. bringing sharks on board and not removing hooks. Line class was not tested as a factor in the analyses because drag weight was not standardized.

#### Generalized additive models

Generalized additive models (GAMs) were used to investigate which factors (fork length, SST and hooking location) influenced the length of fight time and to test the relationship between the characteristics of capture (namely: SST, fight time, handling time, whether sharks were handled on deck, hook type and hooking location) and blood-based dependent variables representing the physiological stress response.

Cleveland dotplots and boxplots were visually inspected to check for outliers in accordance with methods recommended by Zuur *et al.* (2010). Covariates were selected based on correlation matrices, Pearson's coefficients and variance inflation factors. Final selection of covariates for the model was made logically within these constraints (Zuur *et al.*, 2009, 2010; Zuur, 2012). Extreme data need to be removed prior to analysis in order to reduce the likelihood of type 1 and 2 errors. As such, sharks with fight times >70 min (122–513 min) were omitted from GAMs to prevent the clustering caused by extreme values contributing to statistical errors; beyond this time point, relationships become based on too few data points to be considered reliable (plots of the full data are included in Supplemental Figure S1 to illustrate this point).

Beginning with a fully factored model for each response variable, a stepwise, backwards elimination method was used to drop predictor variables from the model based on statistical significance and relevance until only significant predictors remained (Ambelu *et al.*, 2014). A smoothing function was applied to the primary non-linear predictor (fight time or fork length) and the number of knots (inflection points) adjusted so that the spline did not indicate over-fitting (Ambelu *et al.*, 2014). All GAMs were run using the Gaussian family algorithm and Identity link function.

## Results

Thirty-three shortfin mako sharks ranging between 110 and 265 cm fork length (equating to 13.4 and 191.5 kg based on the length–weight conversion presented by Stevens, 1984) were sampled; 23 sharks were caught adjacent to Tas, three adjacent to SA and seven adjacent to NSW. There was no significant difference between the size frequency compositions of sharks from each region (Kruskal–Wallis test: *H* = 2.190, d.f. = 2, *n* = 33, *P* = 0.335).

**Figure 1:**
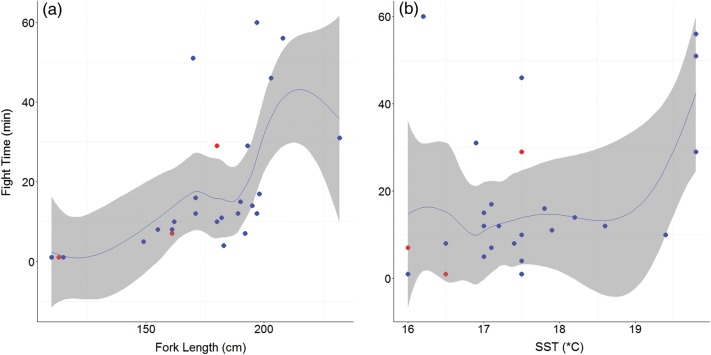
Loess smoothing functions (blue lines) showing the relationship and 95% confidence intervals (grey shading) between fork length (in centimetres) and fight time (in minutes; **a**) and sea surface temperature (in degrees Celsius) and fight time (**b**) for all sharks with fight times up to 70 min (*n* = 29). Tagged individuals (*n* = 26) are overlayed on the function, with blue dots representing survivors and red dots indicating mortalities.

Fight times ranged from 1 to 513 min. A significant relationship existed between fight time and fork length (*F* = 15.862, *P* < 0.0004) and between fight time and SST (*F* = 4.166, *P* = 0.027; *n* = 29, generalized cross-validation score = 169.43, adjusted *r*^2^ = 0.496, deviance explained = 54.8%). Larger sharks had longer fight times, and these times were extended further at warmer SSTs (Fig. 1).

### Hook type and hooking location

Of the sharks sampled, 18 (54.5%) were caught using circle hooks and 15 (45.5%) using J hooks. The majority of sharks caught using circle hooks were jaw hooked (83.3%), whereas the use of J hooks resulted in more variable hooking locations and a significantly lower proportion of sharks hooked in the jaw (20%; χ^2^ = 13.237, d.f. = 1, *P* = 0.0001; Table 2). Only one shark was observed to have substantial bleeding; it was caught using a J hook, which was lodged in the gills. We were able to remove hooks from 12 sharks before release (Table 3).

**Table 2:** Summary of anatomical hooking locations for 33 shortfin mako sharks caught on two types of terminal tackle

Location	J hook (% of hook type in location)	Circle hook (% of hook type in location)
Jaw	3 (20%)	15 (83.3%)
Throat	5 (33.3%)	2 (11.1%)
Gut	4 (26.7%)	0 (0%)
Body	0 (0%)	1 (5.6%)
Gills	3 (20%)	0 (0%)
Total	15 (100%)	18 (100%)

J hooks are 9/0 stainless-steel ‘Shogun’ hooks; circle hooks are 13/0 ‘Eagle Claw’. Numbers and percentages are shown.

**Table 3:** Capture variables for all caught and released shortfin mako sharks

Shark	Size: fork length (cm)	Weight (kg)	Sex	Fight time (min)	Handling time (min)	Hook type	Hook location	Catch condition	Bleeding	Swim off	Displacement (km)	Survived	Blood sample
M007	190	49.8	M	15	5^a^	J	Throat	Good	No	Slow	1042.0	Yes	No
M008	180	59.2	F	29	6^a^	J	Throat	Good	No	Strong	22.8	No (Sitter)	No
M009	192	50.7	M	7	8	Circle	Jaw^b^	Good	No	Well	1597.0	Yes	Yes
M010	189	66.4	M	12	5^a^	Circle	Throat^b^	Good	Unknown	Strong	1386.0	Yes	Yes
M012	161	42.2	F	7	4	Circle	Jaw^b^	Good	No	Well	21.3	No (Sitter)	No
M013^c^	197	77.8	F	266	2	J	Gut	Poor	Unknown	Lifeless	1350.0	Yes	Yes
M014	170	49.8	M	51	3	Circle	Body	Good	No	Well	121.0	Yes	Yes
M015	180	59.2	F	10	2	Circle	Jaw	Good	No	Well	1260.0	Yes	Yes
M016	208	91.8	N/A	56	4	Circle	Jaw	Good	No	Strong	1818.0	Yes	Yes
M017	193	74.3	M	29	3	Circle	Jaw	Good	No	Strong	52.5	Yes	Yes
M018	171	50.7	F	12	7	Circle	Jaw^b^	Good	No	Strong	1671.0	Yes	Yes
M019^c^	240	141.7	F	122	12	Circle	Jaw^b^	Good	No	Strong	47.9	Yes	Yes
M020	149	33.4	F	5	2^a^	Circle	Jaw	Good	No	Strong	344.1	Yes	Yes
M021	110	13.4	F	1	2^a^	Circle	Jaw^b^	Good	No	Strong	132.5	Yes	Yes
M022	183	62.2	M	4	2	J	Throat	Good	No	Strong	79.1	Yes	Yes
M023	115	15.3	F	1	2	J	Throat	Good	No	Strong	427.6	Yes	Yes
M024	162	43.0	F	10	2	J	Jaw	Good	No	Strong	285.9	Yes	Yes
M025	161	42.2	M	8	2	J	Gut	Good	Unknown	Well	392.8	Yes	Yes
M026	113	14.5	F	1	4^a^	J	Gills	Poor	Yes	Strong	0.4	No (Sitter)	Yes
M027^c^	182	61.2	F	160	4	J	Throat	Poor	No	Lifeless	498.3	Yes	Yes
M028^c^	265	191.5	F	513	12	J	Throat	Good	No	Well	1711.0	Yes	Yes
M029	110	13.4	F	4	3^a^	Circle	Jaw^b^	Good	No	Well	No Tag	Unknown	Yes
M030	232	127.8	F	31	10	J	Gills	Average	No	Well	1128.0	Yes	Yes
M031	197	77.8	F	12	3	Circle	Throat	Good	Unknown	Well	1918.0	Yes	Yes
M032	198	79.0	M	17	3	Circle	Jaw	Good	No	Well	756.8	Yes	Yes
M033	120	17.4	M	2	2	J	Jaw^b^	Good	No	Well	No Tag	Unknown	Yes
M034	197	77.8	F	60	4	Circle	Jaw^b^	Good	No	Strong	194.9	Yes	Yes
M035	203	85.2	M	46	5	Circle	Jaw^b^	Good	No	Strong	360.6	Yes	Yes
M036	182	61.2	M	11	3	J	Gills	Average	No	Slow	341.9	Yes	Yes
M037	132	23.2	F	1	1	Circle	Jaw^b^	Good	No	Well	No Tag	Unknown	Yes
M038	171	50.7	F	16	2	Circle	Jaw^b^	Good	No	Slow	318.3	Yes	Yes
M039	195	75.4	F	14	2	J	Jaw	Good	No	Well	530.4	Yes	Yes
M040	155	37.6	M	8	2	J	Gills	Good	No	Well	1652.0	Yes	Yes

Sharks were caught on either 9/0 stainless-steel ‘Shogun’ J hooks or 13/0 ‘Eagle Claw’ circle hooks. ‘Bleeding unknown’ is for deep-hooked sharks where the puncture location was not visible. ‘Displacement’ is the distance (in kilometres) from the release location after 30 days.

^a^Sharks were brought on deck.

^b^Hooks were removed before release.

^c^Sharks that were omitted from generalized additive models.

### Post-release survival

**Figure 2:**
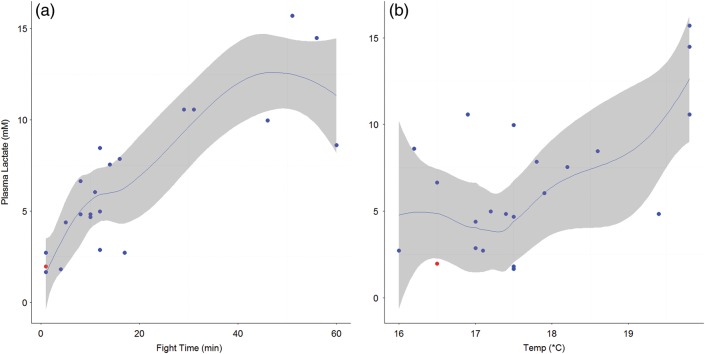
Loess smoothing functions (blue lines) showing the relationship and 95% confidence intervals (grey shading) between calculated plasma lactate (millimolar) with fight time (*n* = 25; in minutes; **a**) and calculated plasma lactate (millimolar) with sea surface temperature (in degrees Celsius; *n* = 25; **b**). Tagged individuals (*n* = 22) are overlayed on the smoothing function, with blue dots representing survivors and red dots indicating mortalities.

**Figure 3:**
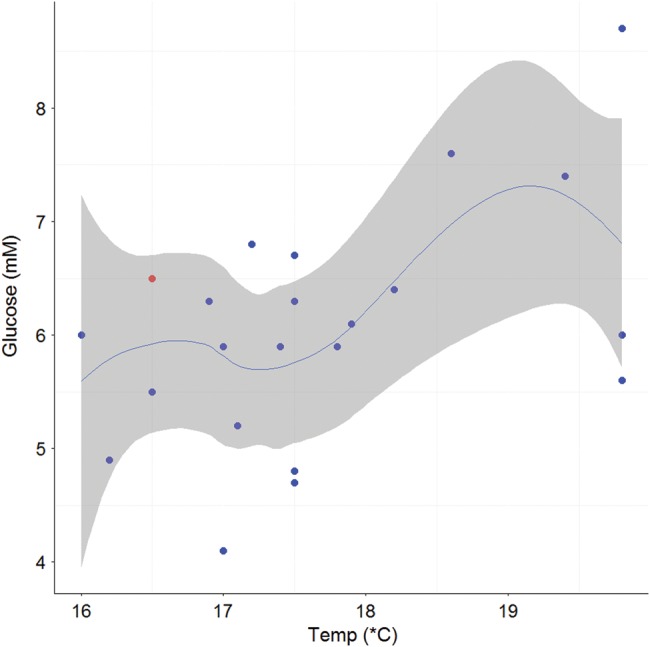
Loess smoothing function (blue line) showing the relationship and 95% confidence intervals (grey shading) between plasma glucose (millimolar; *n* = 25) and sea surface temperature (in degrees Celsius) for all sharks with fight times up to 70 min. Tagged individuals (*n* = 22) are overlayed on the smoothing function, with blue dots representing survivors and red dots indicating mortalities.

**Figure 4:**
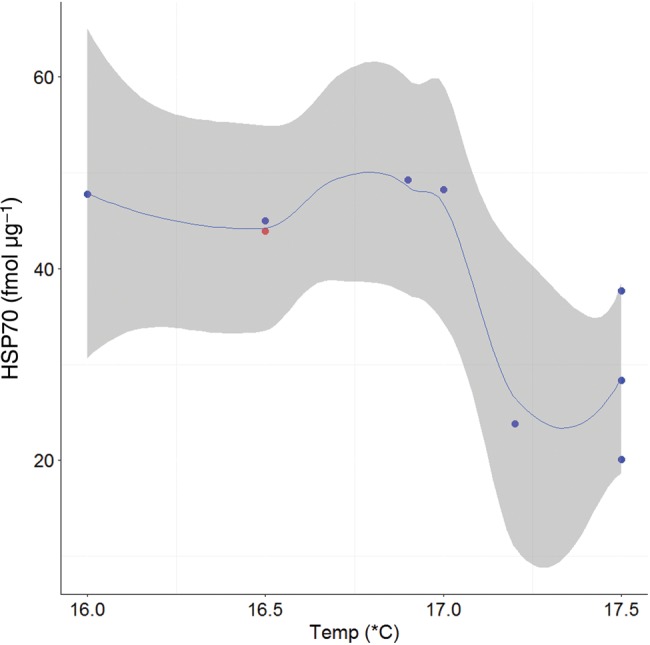
Loess smoothing function (blue line) showing the relationship and 95% confidence intervals (grey shading) between red blood cell heat shock protein 70 (HSP70; in femtomoles per microgram) and sea surface temperature (in degrees Celsius; *n* = 10) for all sharks with fight times up to 70 min. Tagged individuals (*n* = 9) are overlayed on the smoothing function, with blue dots representing survivors and red dots indicating mortalities.

**Figure 5:**
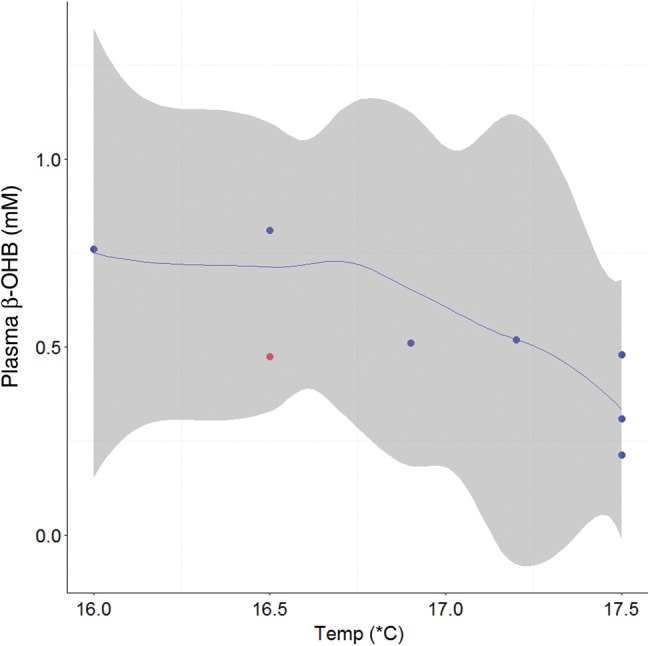
Loess smoothing function (blue line) showing the relationship and 95% confidence intervals (grey shading) between plasma β-hydroxybutyrate (β-OHB; millimolar) and sea surface temperature (in degrees Celsius; *n* = 9) for all sharks with fight times up to 70 min. Tagged individuals (*n* = 8) are overlayed on the smoothing function, with blue dots representing survivors and red dots indicating mortalities.

Thirty sharks were tagged with sPAT tags, 27 of which survived for the full duration of the 30 day tag deployment (Table 3), equating to a survival rate of 90% (95% confidence interval: 80–97%). The three mortalities occurred within 24 h of release and were all categorized as ‘sitters’, meaning that minimal and maximal daily depth were the same, and remained constant for over 24 h. In all instances, these resting depths corresponded with bathymetry, confirming that the shark was resting on the seabed. With the exception of three individuals, the sharks were generally in good condition when captured, and most swam off well (Table 3). Three individuals were, however, in poor condition at release; two appeared moribund and lifeless and the other exhibited severe bleeding; only the latter of these three did not survive. A blood sample was available for only one of the sharks that died, and thus we were unable to investigate the relationship between physiological stress and post-release mortality. Physiological parameters for this shark were, however, well within the ranges of all surviving individuals (Figs 2–5). Although two of the three mortalities were sharks that were brought on board, this handling practice did not have a significant impact on mortality (*P* = 0.094), nor did failure to remove hooks before release (*P* = 0.672).

### Physiological response to capture

Twenty-seven of the tagged sharks were blood sampled, along with three non-tagged sharks. All 30 blood samples were analysed with field meters at the time of capture, with 13 of the frozen blood samples further analysed in the laboratory. None of the tested physiological variables (Table 4) were significantly related to handling time, handling on deck, hooking location or hook type.

**Table 4:** Physiological parameters measured in blood of shortfin mako sharks

Parameter	Minimum	Maximum	Mean	SEM	*n*
Lactate (mM)^a^	0.6	33.8	8.4	1.5	29
Glucose (mM)	4.1	8.7	6.0	0.2	29
Haematocrit (%)	22.5	40	33.8	0.8	26
Plasma Na^+^ (mM)	242	272	252.6	2.7	11
Plasma K^+^ (mM)	3.4	4.4	3.9	0.1	11
Plasma Cl^−^ (mM)	222	240	230.1	1.4	11
Plasma urea (mM)	306.7	399.6	353.1	9.1	11
RBC urea (mM)	237.1	337.5	284.5	9.1	12
Plasma TMAO (mM)	97.5	195.5	139.9	7.3	11
Ratio of urea to TMAO	1.7:1	3.5:1	2.6:1	0.15	11
β-OHB (mM)	0.212	0.910	0.567	0.06	11
RBC HSP70 (fmol µg^−1^)^b^	3.05	49.23	36.6	3.9	12

All parameters measured in millimoles per litre with the exception of haematocrit and RBC HSP70. Abbreviations: HSP70, heat shock protein 70; β-OHB, β-hydroxybutyrate; RBC, red blood cell; TMAO, trimethylamine oxide.

^a^Values as a proxy for plasma lactate calculated from Lactate Pro values.

^b^Values are reported in femtomoles of HSP70 per microgram of soluble protein from the RBCs.

For sharks with fight times up to 70 min, there were significant positive relationships between plasma lactate and increasing fight time (Fig. 2a) and SST (Fig. 2b) and plasma glucose and increasing SST (Fig. 3). There were significant negative relationships between both RBC HSP70 (Fig. 4) and plasma β-OHB (Fig. 5) and increasing SST (Table 5).

**Table 5:** Results from generalized additive models examining the physiological response in sharks with fight times up to 70 min

Model	Predictors (best model)	GCV	Adjusted *r*^2^	Devience explained (%)	*n*	e.d.f.	*F*	*P*-value
Plasma lactate	s (FightTime, k3) s(SST, k3)	3.423	0.820	84.1	25	1.717 1.000	32.210 9.502	<0.0001 0.005
Glucose	s (SST, k3)	0.935	0.141	17.7	25	1.000	4.954	0.035
HSP70	s (SST, k3)	79.526	0.486	56.6	10	1.390	6.701	0.023
β-OHB	s (SST, k3)	0.036	0.474	54.0	9	1.000	8.220	0.023

Only best significant models resulting from the backwards elimination approach are presented. Abbreviations: e.d.f., estimated degrees of freedom; GCV, generalized cross-validation score; HSP70, heat shock protein 70; β-OHB, β-hydroxybutyrate; ‘s’ indicates that a smoothing function is applied to the predictor variable. The value after ‘k’ is the number of knots used in the smoothing function.

Haematocrit and concentrations of plasma Na^+^, plasma K^+^ or plasma Cl^−^, urea (plasma and RBC), plasma TMAO and the ratio between plasma urea and plasma TMAO were not explained by any of the factors tested in our models.

Although not included in GAMs, sharks with fight times in excess of 70 min had significantly higher plasma lactate (*H* = 4.904, *P* = 0.026) and plasma Na^+^ concentrations (*H* = 4.541, *P* = 0.033) than sharks caught within 70 min. No other blood parameters differed significantly between the two fight time groups.

## Discussion

This is the first study to assess post-release survival and capture stress physiology directly for shortfin mako sharks in a recreational fishery. Overall, post-release survival rate is 90% when shortfin mako sharks are caught on rod and reel and subjected to fight times up to 513 min and handled for up to 12 min. The high survival rate supports the efficacy of catch and release as a strategy promoting responsible fishing for this species. Plasma lactate concentrations indicate that substantial anaerobic activity was associated with resisting capture; however, no sign of disruption to ionic or osmotic or energetic homeostasis was observed with fight times up to 70 min. The limited number of sharks with fight times in excess of this threshold precludes conclusions being made about the stress response to longer angling events; although sharks with long fight times did have significantly higher plasma La^−^ and plasma Na^+^ concentrations. These individuals also survived, implying some degree of resilience to the increased physiological impacts of longer fight times. Heat shock proteins were elevated in cooler SSTs; a phenomenon that we believe may be linked to the thermal strategy of this species. Additionally, changes in plasma glucose and plasma β-OHB concentrations were noted with varying SSTs. Three mortalities occurred after short fight times that were not expected to provoke a strong stress response; however, two of these sharks were foul hooked. Taking this into account with the apparent resilience to capture stress, it is most likely that physical injury associated with hook choice had the greatest impact on survival in this study.

### Physiological response to stress

We report an increase in plasma lactate with both fight time and SST; a relationship that has been previously noted in rainbow trout (Kieffer *et al.*, 1994; Meka and McCormick, 2005) but until now, not for elasmobranchs, nor in any aquatic endothermic species. Increases in lactate concentration with fight time alone have been observed in game fish by numerous authors and represent the most consistently reported physiological marker of exhaustive anaerobic activity (Hoffmayer and Parsons, 2001; Heberer *et al.*, 2010; Gallagher *et al.*, 2014).

Increased blood lactate is one of many physiological changes associated with exhaustive exercise, although it is not thought to be linked directly to survival (Wood *et al.*, 1983; Frick *et al.*, 2010). Nevertheless, some authors have observed significant differences in blood lactate concentrations between sharks that they have classed as either moribund or survivors (Hight *et al.*, 2007; Marshall *et al.*, 2012). Marshall *et al.* (2012) recorded significantly higher lactate concentrations in longline-caught (soak time 4–12 h) shortfin mako sharks that they classed as moribund (34.3 ± 5 mM) vs. those that were classed as survivors (16.7 ± 12 mM); however, they did not examine post-release survival directly, nor qualify how they classed animals as moribund. Hight *et al.* (2007) classed longline-caught sharks as moribund based on physical appearance and responsiveness. Likewise, we report lactate concentrations in sharks that are comparable to those assessed as moribund by Hight *et al.* (2007) and Marshall *et al.* (2012) and note that, despite these lactate concentrations and the moribund appearance of sharks boat-side, these individuals survived. For example, the highest recorded lactate value (33.8 mM) was taken from a shark that was retrieved after a fight of >4 h; this individual became tangled in the line and was retrieved tail first, appearing lifeless boat-side, with no active swimming observed at release. This individual recovered, and the tag was detected 30 days later, 1350 km from its deployment location. These findings demonstrate that neither plasma lactate concentration nor subjective physical measures (appearance, responsiveness) are reliable predictors of mortality.

Concentrations of plasma ions and haematocrit were comparable to those of longline-caught shortfin mako sharks (Marshall *et al.*, 2012). Changes in osmolarity and subsequent increases in haematocrit may accompany the onset of acidosis (Turner *et al.*, 1983; Cliff and Thurman, 1984; Skomal and Mandelman, 2012); however, we found no relationship between the variables included in our models and haematocrit or the concentrations of plasma ions. The results relating to the relationship between fight time and plasma ions are likely to be limited by the sample size of sharks used in the GAMs (*n* = 9) and the four sharks with fight times over 70 min being omitted from this analysis. This is supported by the significantly higher distribution of plasma Na^+^ concentrations that we observed in these four sharks relative to the others and suggests that longer fight times may still provoke an ionic response to capture stress in this species.

The osmolytes urea and TMAO are a key component of the elasmobranch osmoconformation strategy (Yancey and Somero, 1979; Treberg *et al.*, 2006). This is the first study to examine TMAO in an endothermic shark species, with plasma concentrations in the shortfin mako shark (139.9 ± 7.3 mM) at similar levels to those measured in ectothermic species such as Port Jackson sharks (*Heterodontus portusjacksoni*; mean = 121 mM; Cooper and Morris, 1998). Our values for plasma urea (353 ± 9.1 mM) were comparable to other reports for this species following angling (322 mM; *n* = 2; Wells *et al.*, 1986) and levels reported for ectotherms such as Port Jackson (394 mM) and gummy sharks (*Mustelus antarcticus*; 377 mM; Frick *et al.*, 2010). There have been reports of significant decreases in plasma urea in response to otter-trawl capture and transport in spiny dogfish (Mandelman and Farrington, 2007) and gillnet capture in gummy sharks (Frick *et al.*, 2010), which are likely to be attributable to a stress-induced increase in gill surface area and urea permeability (Evans and Kormanik, 1985). However, we did not observe any relationship between fight time and either osmolyte; nor did we find a significant difference in the concentrations of these osmolytes between sharks included in our model and those with long fight times. These findings support those of Brill *et al.* (2008), who reported no difference between the plasma urea levels of control and exercise-stressed sandbar sharks (*Carcharhinus plumbeus*). The utility of urea and TMAO as indicators of the stressed state in elasmobranchs remains unclear, as also concluded by Skomal and Mandelman (2012).

The ketone, β-OHB, plays an important role in the supply of energy for exercise recovery, with its oxidation supplying ~20% of the ATP required by *S. acanthias* (Richards *et al.*, 2003). It is also an important energy source for the heart and red muscle in elasmobranchs (Ballantyne, 1997) and therefore plays an integral role in exercise physiology. We observed no relationship between plasma β-OHB concentrations and fight time, which may be explained by the white muscle uptake of plasma ketones occurring at the same rate as their supply from the liver (Richards *et al.*, 2003). We did find a significant negative relationship between β-OHB and SST for sharks caught within 70 min; however, the reason for this relationship is uncertain. The β-OHB concentrations in this study (0.567 ± 0.06 mM) are comparable with those previously reported for mako sharks (0.978 mM; Watson and Dickson, 2001), and the lack of exceptionally high values (∼5 mM) would suggest that none of the sharks in this study was affected by starvation events (Walsh *et al.*, 2006; Wood *et al.*, 2010). Assuming that starvation can contribute to poor health and negatively impact the energy available for metabolic processes such as swimming and recovery, our β-OHB data suggest that the sharks in the present study were not starved or energy depleted. We also observed a positive relationship between blood glucose and SST, with increased blood glucose possibly reflecting an increase in metabolic rate associated with the warmer SSTs and potentially an increase in feeding frequency that is necessary to sustain these increased energetic demands (Hoffmayer *et al.*, 2012).

When all cells experience protein-damaging stress, HSPs are up-regulated within minutes to facilitate recovery of protein structure by guiding refolding, preventing protein aggregation and targeting irreparable proteins for destruction (Roberts *et al.*, 2010; Currie, 2011). We examined the impacts of fight times of up to 70 min on cellular function by quantifying one of the most highly conserved of the stress proteins, HSP70 (Roberts *et al.*, 2010; Currie, 2011). No relationship between RBC HSP70 and fight time was found, although interestingly, we found a significant negative relationship between RBC HSP70 and SST. This is a surprising relationship and one that conflicts with what is known about the heat shock response in teleosts; specifically, that HSP70 usually increases at warmer temperatures (Currie, 2011). Given that the shortfin mako is an endothermic elasmobranch (Bernal *et al.*, 2001) capable of maintaining body temperatures 7–10°C above ambient (Carey and Teal, 1969), the higher RBC HSP70 levels in cooler waters may be a response to a larger temperature difference experienced by the blood as it circulates from the cool periphery to the warm core of the fish. Additionally, increased HSP70 expression may reflect an elevated metabolic rate in response to the cold, as observed in the splenic tissue of Pacific bluefin tuna (*Thunnus orientalis*; Mladineo and Block, 2009). Skomal and Bernal (2010) presented data showing HSP70 expression in shortfin mako sharks with 30–45 min fight times to be six times higher than in those on the line for <1 min; however, the use of relative HSP70 levels and the absence of temperature data presented by these authors prevent any direct comparisons being made with our findings. It is possible that the strong relationship we observed between RBC HSP70 and SST dominated any effects that fight time may have had at a constant temperature. Further research is certainly needed to understand how endothermic fishes use HSPs during stress events and in varying environmental conditions.

### Effect of fight time

Decreased survival associated with long fight times was not observed in the present study, differing from previous reports on pelagic sharks in both commercial (Campana *et al.*, 2009; Gallagher *et al.*, 2014) and recreational fisheries (Heberer *et al.*, 2010). Growth, digestion and exercise recovery all require the delivery of oxygen and metabolic substrate to the tissues at rates above those required by routine activities (Brill, 1996). Hence, elasmobranchs with a high aerobic scope should be capable of supplying more oxygen to tissues to deal with multiple aerobic demands (such as swimming and recovering from stress) simultaneously. This may also enable them to cope with a greater magnitude of physiological disruption from exercise and recover faster from this relative to their less active counterparts (Priede, 1985; Brill, 1996; Sepulveda *et al.*, 2007; Skomal and Bernal, 2010).

Heberer *et al*. (2010) identified fight time as a significant predictor of survivorship for tail-hooked common thresher sharks (*Alopias vulpinus*), with all sharks on the line ≥85 min succumbing to mortality. However, the capture method used by Heberer *et al.* (2010) involved pulling sharks backward, preventing effective ram ventilation and, in turn, limiting the aerobic capacity of the common thresher sharks. In contrast, our results indicate that all individuals with fight times >85 min (*n* = 4, maximum 513 min) survived. As it is unlikely that respiration was inhibited by our capture method, oxygen delivery was not limited and it is probable that the shortfin mako's ability to cope with multiple aerobic demands was not compromised. Moreover, the three mortalities that we did observe had fight times <30 min, suggesting that mortalities in this study were not likely to be a direct consequence of the physiological impacts of fight time and indicate that this species may be more vulnerable to physical damage resulting from gear use and handling.

### Effect of gears

Our data indicate a much higher occurrence of foul hooking associated with the use of J hooks compared with circle hooks. Foul hooking has been shown to increase mortality rate significantly in a number of species (Bartholomew and Bohnsack, 2005; Reeves and Bruesewitz, 2007; Campana *et al.*, 2009; Epperly *et al.*, 2012; Kneebone *et al.*, 2013) and, for the shortfin mako in particular, foul-hooked sharks were more than four times more likely to be retrieved from longlines dead than jaw-hooked sharks (Epperly *et al.*, 2012). Two of the three mortalities observed in the present study were foul-hooked sharks caught using J hooks. One was gill hooked, with the associated bleeding almost certainly the cause of death; the other was deep hooked, possibly sustaining internal injuries or bleeding. Necropsy has shown foul hooking to be associated with hook penetration of the pericardium (Kneebone *et al.*, 2013) and vital organs such as the heart, liver and parts of the lower alimentary canal (Caruso, 2000; Borucinska *et al.*, 2001, 2002). Retained hooks can also lead to mortality over longer periods by causing systemic diseases (Borucinska *et al.*, 2001; Adams *et al.*, 2015). The significant reduction in foul hooking that we observed with circle hook use is in agreement with the findings of many other authors (Caruso, 2000; Cooke and Suski, 2004; Bartholomew and Bohnsack, 2005; Mapleston *et al.*, 2008; Pacheco *et al.*, 2011). It should be noted that although circle hooks are better for fish welfare in the majority of instances, offsetting circle hooks can counteract their conservation benefits by increasing deep hooking and subsequent mortality (Cooke and Suski, 2004; Epperly *et al.*, 2012; Rice *et al.*, 2012). The circle-hooked shark that died in the present study was hooked in the jaw and appeared to be in good health; however, no blood sample was taken and therefore we cannot speculate on the reasons for this mortality beyond the increased risk of predation during recovery.

### Resilience to capture stress

Other survival estimates published for this species relate to individuals mostly taken on longlines and are based on tag-recapture data (79%; Wood *et al.*, 2007) and estimates from quantifying catecholamines at release (80%; Hight *et al.*, 2007). The lower survival estimates presented by these studies are likely to reflect the differences in capture and handling techniques between commercial and recreational fisheries; a finding consistent with blue shark (*Prionace glauca*) hooking mortality between the two sectors (Campana *et al.*, 2006). When compared with other active sharks that exhibit a physiological response to capture of similar magnitude, the shortfin mako, and other lamnids, have an apparently high level of survivorship (Marshall *et al.*, 2012), indicating a resilience to the physiological stresses of capture.

Previous work suggests that the activity level and ecological classification of elasmobranchs will affect the magnitude of their response to capture (Marshall *et al.*, 2012). Taking into account the data presented by Marshall *et al.* (2012) and the results of the present study, we suggest that the mako shark, a species renowned for its high activity, is resilient to capture stress owing to the metabolic rate and aerobic scope attributed to its endothermy. Other active species, lacking the aerobic scope of endotherms, may differ in their ability to recover from intense exercise whilst simultaneously performing other necessary aerobic processes. As a result, these species may exhibit high mortality rates associated with their limited aerobic capacity; for example, blacktip shark (*Carcharhinus limbatus*; 88% mortality) and dusky shark (*Carcharhinus obscurus*; 81% mortality; Marshall *et al.*, 2012). The data presented by Marshall *et al.* (2012) also suggest that some less active species have relatively high survivorship [e.g. tiger shark (*Galeocerdo cuvier*)] and do not appear to become as physiologically perturbed as active sharks (Marshall *et al.*, 2012; Gallagher *et al.*, 2014). This suggests that less active sharks do not require the aerobic scope associated with endothermy to deal with capture stress; rather, these species avoid substantial physiological perturbation altogether, suggesting divergent strategies in dealing with capture stress between active and less-active species.

### Summary

Fight time did not impact shortfin mako shark survival, despite elevated plasma La^−^ and plasma Na^+^ after long fight times, indicating pronounced metabolic acidosis. This highlights the resilience of this species to capture stress and is likely to reflect the aerobic capabilities associated with endothermy. No other physiological responses were found to be related to the duration of the capture event. Fight times reported in the present study represent those that would be imposed by recreational fishers and give merit to the use of catch-and-release fishing as a conservation method for shortfin mako. Post-release survival in this species is most likely to be impacted by hooking injuries, which can be reduced through the adoption of circle hooks. If sharks are deep hooked, our results indicate that leaving hooks in may be beneficial, rather than risk further internal injury trying to remove them (Bartholomew and Bohnsack, 2005; Kneebone *et al.*, 2013). Furthermore, sharks that appeared moribund when boat-side were observed to make a complete recovery after release, which is an important factor to take into consideration when conducting survivorship studies and when making a decision about whether or not to release an individual. Recent studies have highlighted the highly interspecific nature of the stress response in sharks (Marshall *et al.*, 2012; Renshaw *et al.*, 2012), and species can differ greatly in their ability to cope with physiological disruptions (Renshaw *et al.*, 2012); this may apply particularly when comparing ectothermic and endothermic species. Additionally, the need for fishery-specific assessments may be as important as species-specific assessments where gears and handling techniques are expected to vary between users.

## Supplementary material

Supplementary material is available at *Conservation Physiology* online.

## Funding

This work was supported by the Department of Primary Industries, Parks, Water and Environment, Fishwise Community [grant CG2010/116 to J.M.S.]; Equity Trustees and ANZ Trustees, Holsworth Wildlife Research Endowment [S0020481 to R.P.F.] and the Natural Sciences and Engineering Research Council of Canada [NSERC RGPIN-2014-06177 to S.C.].

## Supplementary Material

Supplementary Data
