# Supplementary material for: High survivorship after catch-and-release fishing suggests physiological resilience in the endothermic shortfin mako shark (Isurus oxyrinchus)
Source: Conserv Physiol. 2015 Sep 30;3(1):cov044. doi: 10.1093/conphys/cov044 (PMC4778490; doi:10.1093/conphys/cov044)
Supplement: Supplementary Data [file cov044supp.zip › cov044supp.docx]

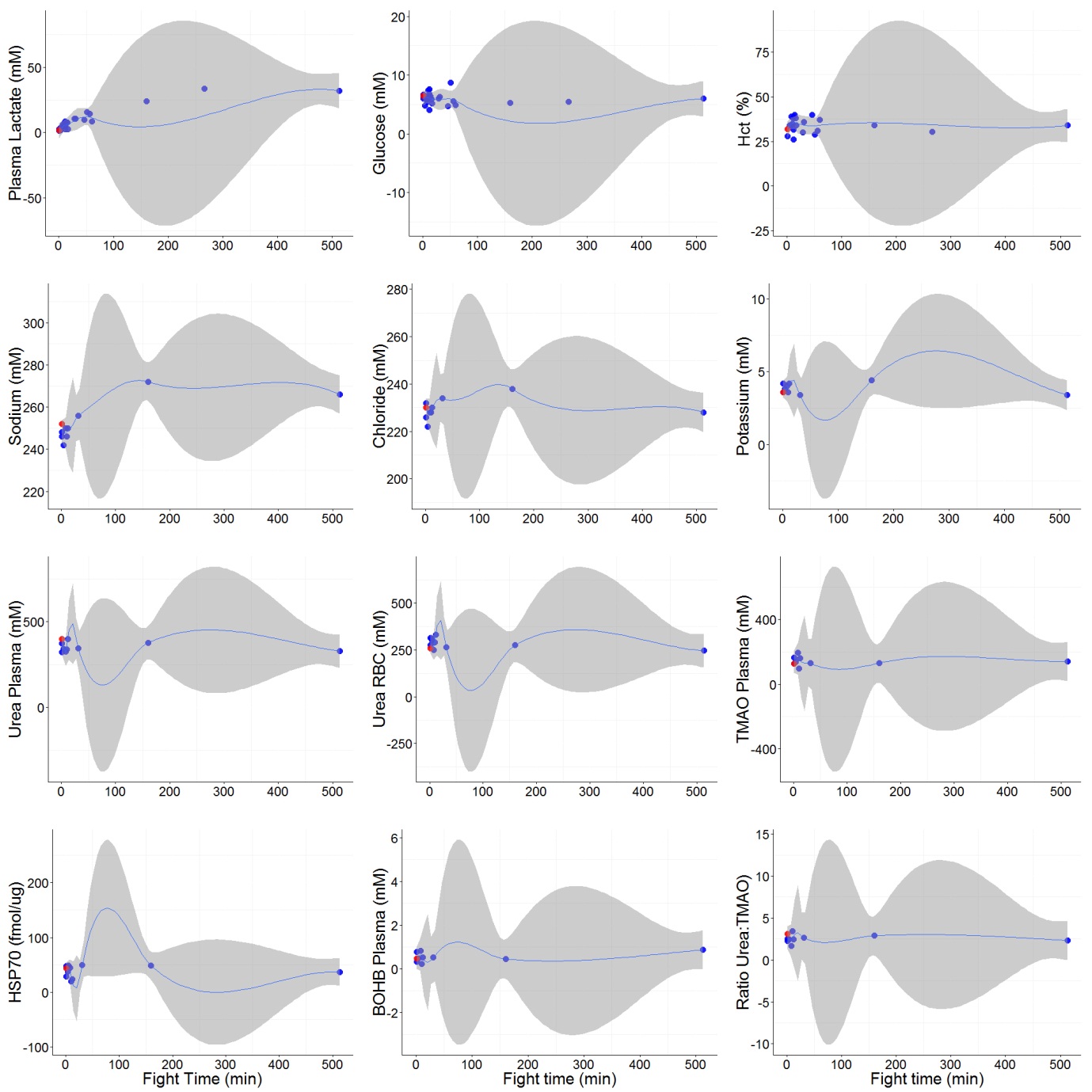


*Supplementary Figure 1: Loess smoothing functions (blue line) showing the relationship and 95% confidence intervals (grey shading) between fight time (minutes) and each of the measured blood parameter all sharks. Tagged individuals (n = 27) are overlayed on the function with blue dots representing survivors and red dots indicating mortalities. The spread of confidence intervals beyond 70 mins illustrates the unreliability associated with including these extreme data in the final GAMs.*
